# Retraction Announcement

**DOI:** 10.12669/pjms.341.15242

**Published:** 2018

The following manuscript has been retracted from May-June 2015 issue on the request of the authors who stated that “after publication, their group found that it was difficult to repeat the results. We believe that there may be some flaws or operational loopholes, hence we would like to retract this paper.”- **Editor**

**Retraction** in: Pak J Med Sci 2015;31(3):672-677. **doi**: http://dx.doi.org/10.12669/pjms.313.7170 **Link**: http://pjms.com.pk/index.php/pjms/article/view/7170

